# Amyloid antibody therapy for early-stage Alzheimer’s disease: a critical review of three recent trials

**DOI:** 10.1007/s00415-024-12361-w

**Published:** 2024-04-13

**Authors:** A. E. Guiloff, P. Rudge

**Affiliations:** 1 Barts and the London School of Medicine and Dentistry, Queen Mary University of London, London, UK; 2 MRC Prion Unit at UCL and UCL Institute of Prion Diseases, London, UK; 3 National Prion Clinic, National Hospital for Neurology and Neurosurgery, London, UK

## Introduction

Pathogenic tau rather than amyloid deposition has been repeatedly reported as a cognitive decline correlate. Monoclonal amyloid-beta antibodies (ABA) target pathological amyloid beta aggregates, one of the multiple proposed drivers of Alzheimer’s disease (AD) pathology. Although clinical trials of ABAs have produced statistically significant outcomes, they are clinically unconvincing. This may reflect the role of tau as a cognitive decline correlate, and the late application of ABAs in AD pathology. As a result, more recent ABA trials are targeting patients at an earlier stage of disease, to limit neurodegeneration before clinical manifestations. However, this is proving problematic as there are a lack of specific biomarkers for detecting true preclinical AD, monitoring treatment response or quantifying disease progression. Despite this, the race for disease modifying therapies in AD is accelerating, with no new approved drugs until the recent initially accelerated FDA approvals of aducanemab and Lecanemab. With blood brain barrier (BBB) antibody penetrance at less than 0.01%, drug doses must be multiplied exponentially for sufficient absorption, creating economic viability concerns. Challenges in AD trials are complicated by a lack of consensus on clinical scoring systems and standards of dementia care. Remaining scientific barriers include incomplete understanding of pathological drivers, and mechanisms for drug delivery.

### Donanemab in Early Symptomatic Alzheimer’s Disease: The TRAILBLAZER-ALZ 2 Randomized Clinical Trial

Donanemab is an IgG1 monoclonal antibody against insoluble truncated pathological beta amyloid. The TRAILBLAZER-ALZ 2 is an 18-month phase 3 trial completed by 1736 participants in 277 sites from June 2020 to November 2021, comparing Donanemab to placebo. Patients were aged 60–85 years, were 95% white, 70% APOE4 carriers and > 50% were on acetylcholinesterase inhibitors prior to recruitment. Patients had early-stage AD with mild cognitive impairment/dementia with amyloid and low/medium/high tau pathology. Patients were dosed 700 mg intravenously for the first 3 doses and 1400 mg thereafter. The primary outcome was the integrated Alzheimer disease rating scale (iADRS) at baseline compared to 76 weeks. Secondary outcome was the clinical dementia rating scale (CDR-SB).

The study reports the Donanemab group had statistically significant reduced clinical progression at 76 weeks. The difference in iADRS (0–144, higher scores indicate worsening) score reduction between Donanemab and placebo was approximately 3 points (*P* < 0.001). The difference in CDR-SB (0–18, higher scores indicate worsening) score reduction was approximately 0.7 points (*P* < 0.001). Amyloid plaques reduced by 88 centiloids (compared to 0.2 in placebo group), with 80% reaching amyloid clearance (< 24 centiloids) at 76 weeks. Least square mean changes in Tau PET were not significant (*P* = 0.97) between Donanemab and placebo groups. The adverse event (AE) profile was significant with 17% of Donanemab patients having life threatening or admission inducing AEs. Deaths considered due to treatment were 3 times higher in Donanemab group, all due to amyloid related imaging abnormalities (ARIA). 37% of Donanemab patients had either ARIA due to oedema (ARIA-E), 75% of which were symptomatic, or ARIA due to haemorrhage (ARIA-H).

*Comment:* The iADRS is out of 144; a ‘meaningful within patient change’ is 9 points for AD patients with mild dementia. A mean difference in improvement of 3 out of 144 is statistically but not necessarily clinically significant. The mean change of CDR-SB was less than 1. The use of mean changes and parametric statistical analysis for a non-linear clinical score (iADRS), assuming normal distribution, should have a reported description of normality for substantiation. A 76-week trial period is also a limited time to assess cognitive changes. Although is an improvement in cognitive decline at 0–24 weeks, amyloid is reported to accumulate intravascularly, this initial improvement could be due to temporary increased cerebral blood flow. Two important, unreported measures are temporal cerebral blood flow measurements and the statistical difference in cognitive improvement at 0–24 and 24–76 weeks.

Furthermore, over 50% of trial participants were on acetylcholinesterase inhibitors, and it is unclear whether these were stopped. Donanemab comparison against the gold standard for these patients would be more accurate, particularly if removal of these was associated with any cognitive worsening in placebo groups. The adverse effect profile of Donanemab is also significant in this trial particularly regarding ARIA. Even if Donanemab slows clinical progression, ARIA and associated clinical phenotypes could be a major impediment to its use. There are no long-term studies of the effects of drug-related ARIA on cognition or disease progression on a damaged brain, nor the effects of removing large quantities of amyloid from the brain, and the function of the remaining tissue.

*John R. Sims et al. JAMA. 2023;330(6): 512–527.*

### Trial of Solanezumab in Preclinical Alzheimer’s Disease

Solenazumab is a monomeric amyloid IgG1 antibody binding to the mid domain of amyloid beta. This randomised double-blind phase 3 trial was conducted across 67 sites from 2014 to 2022. Recruited patients were aged 65–85 years with preclinical AD (clinical dementia rating scores of 0 or MMS > 25 and elevated amyloid levels on f-florbetapir PET). Patients were administered up to 1600 mg solanezumab or vehicle intravenously every 4 weeks for 240 weeks. Primary outcome was the preclinical Alzheimer cognitive composite score, (PACCs, 0–96), a validated scoring system where higher scores indicate clinical improvement. 94% of patients were white and 59% carried at least 1 APOE4 allele.

The study reported the Solenazumab group did not have significantly reduced cognitive decline compared to placebo. Results for mean change were not significant and worse in the solanezumab group for the following parameters: PACCs (− 0.3, *P* = 0.26), CDR-SB score (0.12, higher is worse). The following parameters were not significant and improved in the solanezumab group: CFI combined score, (0.58, lower is worse) ADL partner score (− 0.61, higher is worse). Amyloid levels on PET were increased by 11.6 centiloids in the Solanezumab and 19.3 centiloids in placebo. There was no significant reduction in tau on imaging. AE profiles were minor and ARIA-E occurred in < 1% of participants in each group. ARIA-H was present in 29% and 32.8% of the solanezumab and placebo groups, respectively.

*Comment:* Overall, the study reports no efficacy for solanezumab in reducing clinical cognitive scores across a longer study time of 240 weeks. However, most patients did not show disease progression even within placebo groups. The criteria of no cognitive impairment and amyloid elevation on PET (present in normal ageing) may also not be representative of the pre-clinical Alzheimer’s population. Prodromal Alzheimer’s needs further characterisation and specific, sensitive biomarkers. Solanezumab has a different mechanism and consequent safety profile, with lower ARIA rates and these data may indicate ARIA is more likely to affect later stage confirmed AD patients.

*R.A. Sperlin et al. N Engl J Med. 2023;389:1096–107.*

### Two Phase 3 Trials of Gantenerumab in Early Alzheimer’s Disease (GRADUATE I/II)

Gantenerumab is an IgG1 monoclonal antibody with an affinity for amyloid beta aggregates.

‘GRADUATE’ I/II were 2 randomised double-blind trials with 985 and 980 participants, respectively, in 288 sites across 116 weeks. Patients were aged 50–90 with mild cognitive impairment/mild dementia and amyloid plaques on PET or CSF testing. Patients were randomized to Gantenerumab or placebo, starting at 120 mg with a target dose 510 mg. 62.5% of patients were on Alzheimer’s drugs. The primary outcome was the CDR-SB score.

The study reported no significant slowing of cognitive decline in gantenerumab groups. Change in CDR-SB between placebo and Gantenerumab was − 0.31 (*P* = 0.10) and − 0.19 (*P* = 0.30) in GRADUATE I/II respectively. Amyloid plaques were reduced in Gantenerumab groups as indicated by Amyloid PET, with reductions of 66 (28% < 24 centiloids amyloid negative) and 57 centiloids (27% of patient’s amyloid negative) in GRADUATE I/II, respectively. There was no significant reduction in tau on imaging. CSF analysis showed lower CSF p-tau, and higher AB42 in Gantenerumab groups. Brain volume changes in Gantenerumab groups across both trials reported greater loss of total brain volume and increase in ventricular volume compared to placebo. In the GRADUATE I trial, there was greater decrease in left hippocampal volume in Gantenerumab groups. 25% of patients had ARIA-E (5% total symptomatic). Patient carriers of the homozygous e3 allele of APOE were reported as more likely to have ARIA-E in Gantenerumab groups. Serious adverse events were reported in 14%. Safety follow-ups were completed at 14 and 50 weeks, along with a dose de-escalation scheme.

*Comment*: There was no reduction in cognition in placebo or gantenerumab groups, and the specificity of recruitment criteria for true early AD patients is also unclear. The observation of a reduction in brain volume may not be neurodegeneration but represent fluid shifts following rapid reductions in amyloid. This study used a dose de-escalation scheme and safety follow ups, with reduced symptomatic ARIA-E compared to other studies.

*R. J Bateman et al. N Engl J Med 2023;389:1862–76.*

## Conclusion

Clearance of amyloid accumulation with ABAs has not been shown to reduce long-term cognitive decline in these trials. In all of these studies, reductions in tau, a known cognitive decline correlate in AD, were statistically insignificant on imaging. Within ABA trials, initial cognitive improvement should be correlated with cerebral blood flow measurements to determine the role of vascular amyloid clearance in short term cognitive improvement. Statistical comparisons between initial cognitive improvement and later trial outcomes, aided by longer total trial times, could better quantify true, permanent clinical improvements. Amyloid antibody treatments should be compared to current treatment standards rather than placebo. Care should be taken in declaring positive trial results, including correlation of statistical significance with clinical utility, particularly in trials involving large numbers of patients with little cognitive reserve. This is challenging in AD, with no consensus on standard of care, cognitive outcome measurement and a heterogenous disease course. Negative results remain invaluable in informing future research and learning from prior negative results, the rationale for continued high power trials for similar patient results may require review. The shift in AD trial recruitment from later stage to earlier stage patients highlights a need for specific, sensitive biomarkers to identify true early AD. This is vital in targeting earlier stages of disease progression to halt neurodegenerative pathology before its permanent effects materialise. In addition, there remain issues with patient recruitment including the 90% white demographic, which fails to represent the ethnically diverse patient AD population. Not only were ABAs ineffective in cognitive decline reduction, but their adverse effect profile was significant. Long term analysis of the effects of ARIA, rapid reduction in amyloid volume and functionality of remaining tissue requires further study.

